# Impact of municipal and industrial waste incinerators on PCBs content in the environment

**DOI:** 10.1371/journal.pone.0242698

**Published:** 2020-11-19

**Authors:** Marta Gabryszewska, Barbara Gworek

**Affiliations:** The Institute of Environmental Protection—National Research Institute, Warsaw, Poland; Tsinghua University, CHINA

## Abstract

Polychlorinated biphenyls (PCBs) have been withdrawn from the market due to their toxicity, bioaccumulation capacity, and persistence. PCBs have been observed to potentially form in combustion processes under appropriate conditions and in the presence of precursors containing chlorine. The study covered a municipal waste incineration plant and an industrial waste incineration plant. The objective of the study was to assess the effect of these objects on PCB accumulation in soil and plants taking into account the distance from the emission object and wind direction. Soil samples were collected from layers: 0-5, 5-10, 10-20, and 20-30 cm. Test plants were collected from the same areas as the soil samples. The highest accumulation of PCBs was found in plants with large leaf area. Around the municipal waste incineration plant, these were *Tanacetum vulgare* leaves (12.45 ng/g), and around the industrial waste incineration plant–grasses (4.3 ng/g). In the case of soils, the accumulation of PCBs for both kind waste incinerators was similar, reaching approximately 3 ng/g. As the distance from the municipal waste incinerator and industrial waste incinerator increased, the accumulation of PCBs in the soil decreased. For municipal waste incinerator, no effect of wind direction on PCB accumulation in the soil was observed. In the majority of cases, the accumulation of PCBs in soils taken from the leeward side of the industrial waste incinerator was higher than that in soils from the windward side. In soils around the municipal waste incinerator, PCB compounds moved deep into the soil and reached the highest accumulation in the soil layer of 10-20 cm or 20-30 cm. In soils around the industrial waste incinerator, the highest accumulation of PCBs occurred in the soil layer of 0-5 cm.

## Introduction

Due to the high toxicity of polychlorinated biphenyls (PCBs), their lipophilicity, and bioaccumulation capacity, polychlorinated biphenyls have been taken out of service [1–4]. Small quantities of PCBs can be formed spontaneously from suitable precursors, e.g. during waste incineration [5], drinking water or wastewater chlorination, chlorine bleaching of cellulose pulp [4], or pigment production [6]. Non-ortho PCBs and mono-ortho PCBs may be formed during primary incineration of municipal solid waste under oxidation conditions. Coplanar PCBs are formed as a result of dimerisation of chlorobenzenes during primary incineration. In the process of secondary incineration, a large part of coplanar PCBs is destroyed. Another way of PCBs formation is de novo synthesis [7]. Waste incineration plants, chemical plants, and combined heat and power plants have been determined to constitute the source of incidental PCB production [8–11]. Dibenzenofurans may be formed during the combustion process of PCBs, and if dielectric PCB fluids also contain tri- and tetra-chlorobenzenes, dioxins may also be formed. PCBs are persistent in the environment, and can be transported by air and deposited in the solid or gas phase in water, soil, and plants [12]. Exhaust gases from nine incinerators have been tested in Korea. It was found that, depending on the combustion conditions, large quantities of PCBs with a low level of chlorination, or PCBs with a high level of toxicity may be formed in incineration plants [13]. Injecting biphenyl into the post-combustion zone of the reactor did not change the patterns of emerging PCBs, indicating that the availability of the backbone structure is not a limiting factor for PCB formation [8]. In Taiwan, 3780 fg/Nm PCBs [10] were detected in the air near a solid waste incinerator. Ling and Hou examined PCB content in dust from power plants and incineration plants and obtained the following results: 0.0170 ng/g–oil-fired power plants, 0.033 ng/g–coal-fired power plants, 0.006-0.041 ng/g–solid waste incineration plants, 0.591 ng/g–medical waste incineration plants [14]. In Turkey, average PCB contents of 235, 1.05, and 0.38 ng/g were determined around a cogeneration heat and power plant (CHP) plant in sediment, soil, and ashes, respectively [15]. During the combustion of coal and wood, 8800 ng PCBs/kg of fuel [16] is released into the atmosphere in domestic furnaces. Burning an alternative fuel or shredder residue from old cars results in PCB emission to the atmosphere, with both fuels dominated by the following homologues in the series: tri-CB > tetra-CB > di-CB [17]. In the United Kingdom, the most frequently detected PCB congeners in the exhaust gases from waste incineration plants were 118, 123, and 180 [18]. In laboratory tests, 118 PCB congeners were mainly formed during the combustion of a sample of powdered polyvinyl chloride [19]. PCBs may also be formed during the combustion of biomass. Moltó et al. carried out an experiment involving burning tomato plants in a laboratory reactor at 500°C and in a home furnace at 220°C. The amount of PCBs produced in the exhaust gases was measured, and the following results were obtained for the reactor: 3284.46 ng/kg of biomass and 1.68 ng/kg of burnt biomass for the household stove [20]. The above studies illustrate that despite the lack of PCBs in waste, PCBs can be formed when various wastes are incinerated. By adding a mixture of inhibitors such as (NH_4_)_2_SO_4_, (NH_4_)_2_S_2_O_3_, and (NH_2_)_2_CO + S (1:1) the formation of PCBs can be significantly reduced [21].

PCBs can be transported in the air for long distances and spread almost all over the globe. PCBs from the air is transferred to the ground surfaces via dry particulate deposition [22]. PCBs, due to high octanol-water partition coefficients (ranged from 4.3 to 8.3), are effectively sorbed to soils and sediments [4]. PCBs can also be transported bound to eroded soil or sediments. PCBs are hydrophobic, so they are transported together with a particulate material to the lower levels of the catchment [23]. PCBs sorption onto soil decreased with an increase of humic acids concentrations in soil. [24] It means that PCBs adsorption onto the soils is related to soil pH. The study objective was to examine the impact of a municipal waste incinerator and industrial waste incinerator as a source of PCBs released to the environment on PCB contamination of soils and plants, with consideration of the distance from the incinerators and wind direction. The assessment was based on the spatial and profile distribution of PCBs in soils in the vicinity of the incinerator, and on PCBs contents in plants growing on the aforementioned soils. The impact of incinerators on PCBs accumulation in the environment was assessed by means of calculation of the Bioaccumulation Coefficient (BAC).

## Materials and methods

### Collection of samples

**Fig 1:**
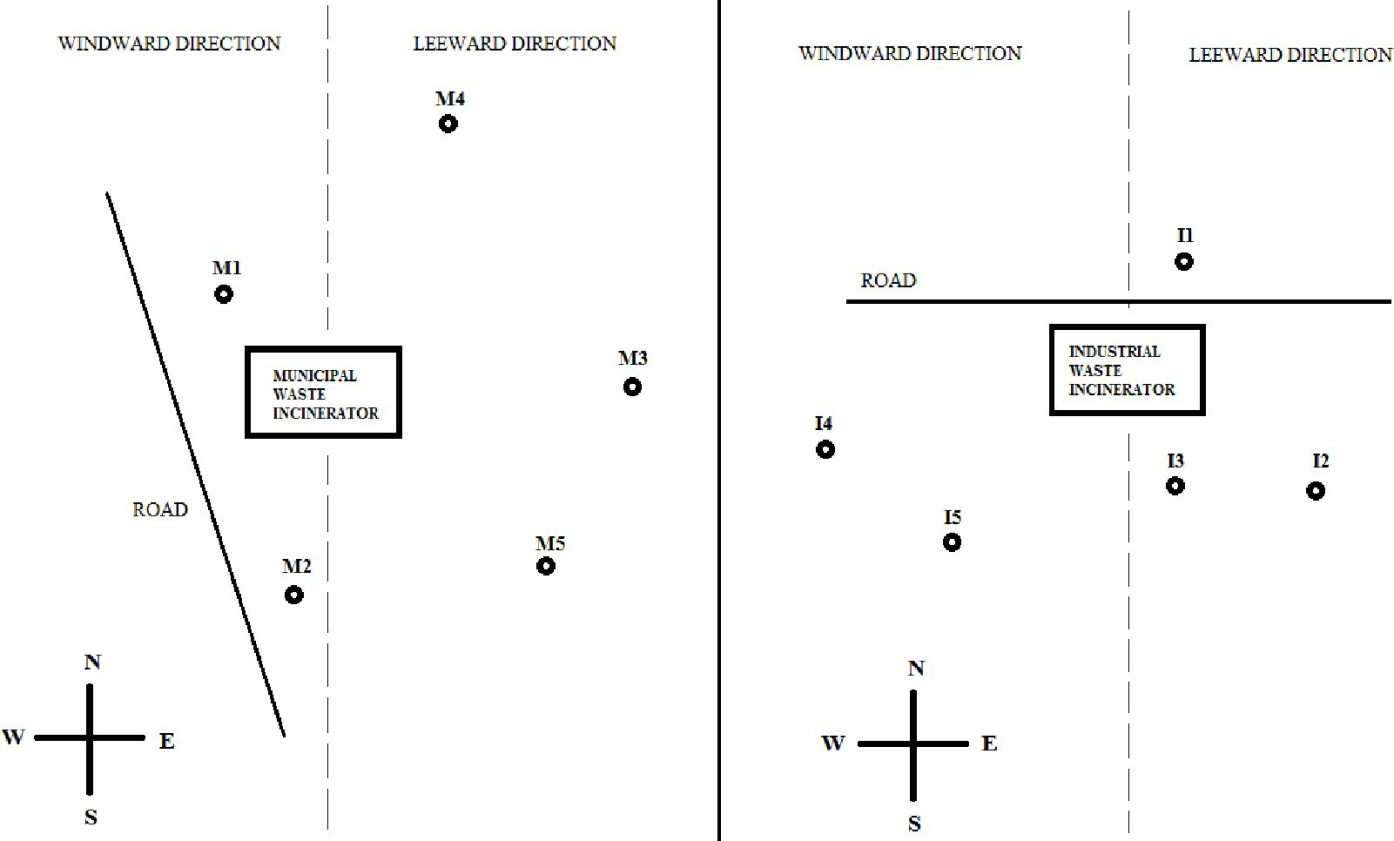
Map of research areas around waste incinerators.

Five study areas were selected for research around a municipal waste incineration plant (in central Poland; 52°13′N, 21°00′E) and industrial waste incineration plant (in southern Poland; 50°20′N, 19°12′E). Samples were taken from abandoned wastelands and no permits were required. These areas were on both the windward and leeward sides. The distribution of the study objects is presented in Fig 1. Soil samples were taken from 0-5, 5-10, 10-20, and 20-30 cm soil layers. In areas I4 and I5, it was only possible to collect soil samples from the 0-5 cm layer. Soil and plant samples were taken from abandoned areas where no agrotechnical treatments were carried out The plant samples were monocotyledonous and dicotyledonous plants collected from the same sites as the soil samples. Leaves of the following tree species were collected for analysis: *Acer negundo*, *Salix sepulcralis*, *Quercus L*., *Populus tremula*, *Jasminum L*., *Cerasus avium*, and *Betula pendula*. For *Impaiens parviflora*, *Poa nemoralis*, and *Utrica dioica*, the entire aboveground part was subject to analysis. *Tanacetum vulgare* was divided into stalks and leaves and flowers together, and *Solidago canadensis* into leaves and stalks. One-year and two-year-old *Pinus L* needles were also included in the study (Table 1).

**Table 1:** List of the plants collected for testing around municipal and industrial waste incinerators.

Plot No.	Plants from the municipal waste incinerator area (abbreviation)	Plants from industrial waste incinerator area (abbreviation)
**1**	*Acer negundo* (An)	*Poaceae* (P)
	*Poa nemoralis* (Pn)	*Pinus L*. (Pi)
	*Tanacetum vulgare* (Tv)	*Populus tremula* (Pt)
**2**	*Acer negundo* (An)	*Jasminum L*. (J)
	*Utrica dioica* (Ud)	
	*Salix sepulcralis* (Ss)	
**3**	*Quercus L*. (Q)	*Poaceae* (P)
	*Impatiens parviflora* (Ip)	
**4**	*Solidago canadensis* (Sc)	*Poaceae* (P)
	*Poaceae* (P)	*Cerasus avium* (Ca)
**5**	*Poaceae* (P)	*Betula pendula* (Bp)
	*Tanacetum vulgare* (Tv)	

The test areas were located at different distances from the incineration plant. Table 2 shows the estimated distances of these areas from the incineration plant chimneys.

**Table 2:** The average distances of test areas from chimneys of municipal and industrial waste incinerators.

Plot No.	The average distance from the test area to the incinerator stack (m)	
	Municipal waste incinerator	Industrial waste incinerator
**1**	103	170
**2**	207	238
**3**	460	148
**4**	500	670
**5**	440	568

### Chemical analysis

The soils for analyses were air-dried at a temperature of approximately 22°C, ground in a mortar, and sieved through 1 mm mesh sieves. The granulometric composition was determined by means of the aerometric method of Casagrande modified by Prószyński, and soil pH–by means of the potentiometric method in H_2_O and 1N KCl. Organic carbon (OC) in the soil was determined on Shimadzu TOC-5000A apparatus at 680°C.

The plants and soils were subject to a freeze-drying process prior to PCBs determination. The soils were then ground in a mortar and sieved through a 1 mm sieve. Contents of PCBs in plants were determined following prior fragmentation. Approximately 15-20 g of dry soil or 5-10 g of dry plant material was extracted in n-hexane (95% pure) using a fast ASE 350 extractor for 20 min in elevated pressure at a temperature of 120°C. The extract was transferred to a flask and concentrated to 1 ml in a vacuum evaporator with a heated bath. The concentrated extract was purified using column chromatography. The columns were filled with florosil (5 cm) and aluminium oxide (5 cm). Gradient washing out was applied, using 20 ml n-hexane and 5 ml mixture of n-hexane: acetone (max. 5% acetone in the mixture). The eluate was concentrated to dry form in a vacuum evaporator with a heated bath. The remaining substance was dissolved in 1 ml n-hexane (GC 99% pure). The resulting analyte was analysed using gas chromatography by means of a Varian electron capture detector (GC/ECD). The substances were separated by means of the VF-Xms column (30 m x 0.25 mm x 0.25 μm). Helium was applied as the carrier gas (purity 5.0; flow 1 ml/min). The temperature sequence in the oven was as follows: 70 ^o^C for 3 min and 70-300°C at a rate of 5°C/min [21]. There were two levels of control of the blank sample (solvent):

- the blank sample went through all the stages of extraction and purification until analysis on the chromatography,
- during the chromatographic analysis, a blank sample was injected between each analyzed sample in order to rinse the column and confirm the lack of interference.

Qualitative analysis of the studied compounds was based on signals (peak surface) using the calibration curve method. The limit of quantification (LOQ) was evaluated for all the analysed compounds. Indicative congeners with expanded uncertainties (U) were determined in the studied samples and expressed in per cent values. The recoveries were calculated for each congener based on testing of soil certified materials and spiked samples for plants. The final result for soils and plants were corrected for recovery values for each congener [25]. The method validation parameters are presented in Table 3.

**Table 3:** Validation parameters.

PCBs congener	28	52	44	99	101	95	110	118	153	105	138	180
**Retention time [min]**	30.153	31.427	32.185	33.748	34.590	34.739	35.938	37.022	37.802	38.220	39.208	41.905
**Linearity: Correlation coefficient R**^**2**^	0.999	0.998	0.996	0.995	0.995	0.995	0.995	0.998	0.995	0.995	0.997	0.997
**LOQ plants (corresponding to lowest level of calibration curve) (ng/g)**	0.02	0.04	0.03	0.03	0.04	0.03	0.03	0.04	0.02	0.04	0.03	0.07
**LOQ soil (corresponding to lowest level of calibration curve) (ng/g)**	0.01	0.01	0.01	0.01	0.01	0.01	0.01	0.01	0.01	0.01	0.01	0.02
**% RSD plants spike samples with 0,1 ng/g n = 12**	13	7	10	8	9	20	30	23	15	22	10	24
**% Recovery of spiked samples plant 0,1 ng/g n = 12**	79	88	64	77	75	95	82	111	120	105	103	125
**% RSD plants spike samples LOQ level n = 6**	15	19	13	17	19	11	15	18	16	13	13	14
**% Recovery of spiked samples plant LOQ level n = 6**	95	56	53	58	51	54	59	51	50	54	53	54
**% RSD certified material soils n = 18**	10	9			10			17	10	10	18	18
**% Recovery of certified material soils n = 18**	81	81			75			72	80	88	122	98
**% RSD soil spike samples LOQ level n = 6**	6	9	16	12	10	11	13	9	10	13	11	15
**% Recovery of soil spike samples LOQ leve n = 6**	120	112	114	116	123	125	116	99	95	100	104	97

### Statistical analysis

Possible relationships between chemical parameters and PCBs content were evaluated by means of regression analysis. All statistical analyses were performed with the application of STATISTICA 9.0 software.

## Results and discussion

### Soil

The soils around the municipal waste incinerator developed from loose sands or weakly clayey sands. The total organic carbon content of the topsoil layers (layer 0–5cm) was between 1% and 22%. Depending on the place of collection, the soils were acidic or slightly alkaline, with pH_KCl_ values ranging from 4 to 8. The soil layers around the industrial waste incinerator developed from clay sands and deeper layers from ordinary or clayey dust. Total organic carbon content in the topsoil layers was approximately 1–4%. The pH_KCl_ values of the soil were in a range of 7.3-8.

In the case of soils around a municipal incinerator, PCBs compounds were observed to move deep into the soil, reaching the highest accumulation in the 10-20 cm or 20-30 cm soil layers. The movement of PCBs deep into the soil was possible due to the loose soil structure. The highest accumulation was reached by congeners: 52, 44, and 110. These congeners belong to homologues with four or five chlorine atoms in the molecule. The presence of larger quantities of PCBs congeners with a small number of chlorine atoms is explained by the fact that such congeners are more easily produced by burning waste or incomplete combustion of petrol than congeners with more chlorine atoms in the molecule. Moreover, congeners with a small number of chlorine atoms in the molecule could be degradation products of congeners belonging to higher PCBs homologues.

**Fig 2:**
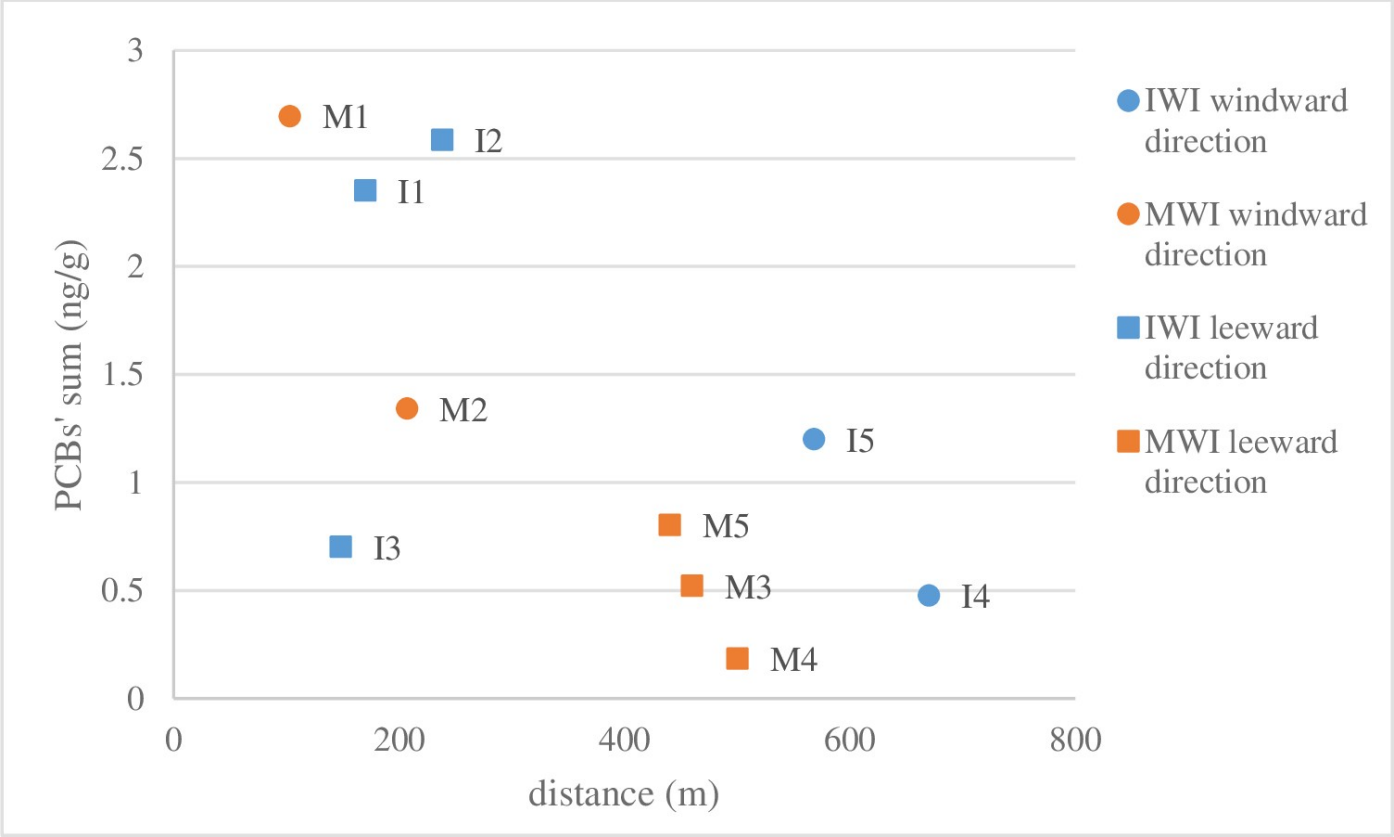
Dependence of PCBs accumulation in the topsoil layer on the distance from waste incinerator plants.

The results for MWI (Table 4) suggest the highest accumulation of PCBs in the soil layer 20-30 cm. Total investigated PCBs in soil are recorded in the following series: total PCBs in M1 > total PCBs in M2 > total PCBs in M5 > total PCBs in M4 > total PCBs in M3. The highest accumulation of all determined PCBs in the topsoil layer (0–5 cm) occurred in the plots closest to the waste incineration plant (Fig 2). The dominant PCBs congeners were 44, 52, and 110. As the distance from the incinerator increased, the accumulation of PCBs in the soil decreased. No effect of wind direction on the accumulation of PCBs in the soil was determined. Test areas M1 and M2 were much closer to the waste incineration plant than M3, M4 and M5 as it was not possible to take samples at the same distance on the windward and leeward sides. Therefore, only the influence of the distance from the incinerator on the accumulation of PCBs in the soil is visible. The highest accumulation of PCBs was determined in soils from study areas M1 and M2. Both these areas were close to the road. Studies on the accumulation of PCBs in soils and plants collected along expressways have shown that road transport is a source of PCBs release to the environment [25].

**Table 4:** PCBs congener content in the soil around municipal waste incinerator (ng/g).

Plot No./depth (cm)	PCBs congeners										
	28	52	101	118	153	138	180	44	105	110	95 + 99
**M1/0-5**	< 0.003	0.167	0.012	0.043	< 0.003	< 0.003	0.061	0.232	0.200	1.982	< 0.003
**M1/5-10**	0.057	0.203	< 0.003	0.019	< 0.003	0.005	0.058	0.137	0.090	0.756	< 0.003
**M1/10-20**	0.087	0.311	< 0.003	0.031	< 0.003	< 0.003	0.089	0.206	0.086	0.727	0.034
**M1/20-30**	0.006	0.381	0.021	0.065	< 0.003	0.021	0.336	0.265	0.352	1.489	0.080
**M2/0-5**	0.028	0.182	0.080	0.168	0.179	< 0.003	< 0.003	0.281	0.086	0.193	0.146
**M2/5-10**	0.018	0.184	0.087	0.101	0.163	< 0.003	< 0.003	0.473	0.091	0.300	0.112
**M2/10-20**	0.029	0.222	0.144	0.107	0.164	< 0.003	< 0.003	0.299	0.131	0.328	0.189
**M2/20-30**	0.010	0.405	0.135	0.065	0.078	< 0.003	< 0.003	0.644	0.053	0.266	0.071
**M3/0-5**	0.010	0.037	0.054	0.056	0.060	< 0.003	0.041	< 0.003	0.037	0.186	0.042
**M3/5-10**	0.012	0.011	< 0.003	0.020	< 0.003	< 0.003	0.054	< 0.003	0.022	0.018	< 0.003
**M3/10-20**	0.012	0.040	< 0.003	< 0.003	0.006	< 0.003	< 0.003	< 0.003	0.022	< 0.003	0.026
**M3/20-30**	0.013	< 0.003	< 0.003	< 0.003	< 0.003	< 0.003	0.021	< 0.003	< 0.003	< 0.003	0.047
**M4/0-5**	0.007	0.044	< 0.003	< 0.003	0.041	< 0.003	0.039	< 0.003	< 0.003	0.040	0.014
**M4/5-10**	0.013	0.031	< 0.003	0.015	0.101	< 0.003	0.033	< 0.003	< 0.003	0.053	< 0.003
**M4/10-20**	0.019	0.042	< 0.003	0.072	0.078	< 0.003	0.021	< 0.003	< 0.003	0.050	< 0.003
**M4/20-30**	0.012	0.090	< 0.003	0.048	0.067	< 0.003	0.015	0.015	0.017	0.042	< 0.003

In soils around IWI, the highest accumulation of PCBs was determined in the 0-5 cm soil layer (Table 5). In the soil’s top layer from study areas I1-I3, PCB 28 congener was dominant, while in soils from research areas I4 and I5, the highest content was reached by congener PCB 52. The compact structure of soils (mainly clay sands) contributed to the limited movement of PCBs into the soil. The highest accumulation of PCBs in the soil’s top layers was determined in profile I2, in the layer of 0-5 cm. Total investigated PCBs content in topsoil is recorded in the following series: total PCBs in I2 > total PCBs in I1 > total PCBs in I5 > total PCBs in I3 > total PCBs in I4. In the majority of cases, PCB content in soils collected on the leeward side was higher than that in soils collected on the windward side. In most cases, the accumulation of PCBs in the soil was higher for samples taken from areas closer to the IWI than for those more remote (Fig 2*)*. In areas, I1 and I2 high PCB 153 contents have been marked. In these areas, trucks have been moving, which indicates that the source of PCB 153 in the soil is transport [25]. In research area I3 there was less accumulation of PCBs than in areas I1 and I2 because there was no additional source of PCBs such as transport. The dominant PCBs congeners belonged to tri-CB, tetra-CB, and penta-CB homologues with a small number of chlorine atoms in the molecule. PCBs compounds with a small number of chlorine atoms in a molecule move more easily in the environment than PCBs compounds with more chlorine atoms. This is because lower chlorinated PCBs are poorly sorbed in soil due to lower values of octanol-water partition coefficients than higher chlorinated PBCs. PCBs compounds with a small number of chlorine atoms in a molecule can be transported over larger distances than PCBs with a larger amount of chlorine atoms, as evidenced by the content in Arctic snow of compounds such as PCB 5, PCB 11, and PCB 52 [26]. Total PCBs content in the top layers of soils around both incinerators had similar values of approximately 3 ng/g. Humic substances are capable of ionic, donor-acceptor, and hydrophobic bonding. Therefore, they can bind POPs in non-toxic and nonbioavailable humic complexes [27]. The higher the soil pH, the lower the PCBs content is in the soil. PCBs content in the soil was determined to depend on soil pH. The lower the soil pH, the lower the PCBs content in the soil.

**Table 5:** PCBs congener content in soil layers around industrial waste incinerators (ng/g).

Plot No./depth (cm)	PCBs congeners										
	28	52	101	118	153	138	180	44	105	110	95 + 99
**I1/0-5**	0.037	0.139	0.034	< 0.003	1.755	< 0.003	0.065	0.114	0.059	0.089	0.061
**I1/5-10**	0.005	0.033	0.027	0.040	< 0.003	< 0.003	0.010	0.019	< 0.003	0.015	< 0.003
**I1/10-20**	0.010	0.068	< 0.003	0.029	< 0.003	< 0.003	< 0.003	< 0.003	< 0.003	0.015	0.016
**I1/20-30**	0.011	0.049	< 0.003	< 0.003	< 0.003	0.061	0.021	< 0.003	< 0.003	0.016	0.006
**I2/0-5**	0.013	0.236	0.035	0.081	1.823	0.048	0.086	0.065	< 0.003	0.160	0.041
**I2/5-10**	0.048	0.117	0.032	0.014	< 0.003	0.044	0.061	0.015	< 0.003	0.171	0.019
**I2/10-20**	< 0.003	< 0.003	< 0.003	< 0.003	< 0.003	0.040	< 0.003	0.039	< 0.003	0.194	0.006
**I2/20-30**	0.014	< 0.003	0.022	< 0.003	< 0.003	0.017	< 0.003	0.017	< 0.003	0.067	0.014
**I3/0-5**	0.011	0.070	0.029	0.054	< 0.003	0.040	0.174	0.027	0.079	0.185	0.034
**I3/5-10**	0.012	0.078	0.019	< 0.003	< 0.003	0.079	0.073	0.015	0.072	0.342	0.031
**I3/10-20**	0.005	0.058	< 0.003	0.011	< 0.003	0.055	0.039	0.006	0.071	0.132	0.035
**I3/20-30**	0.004	0.016	< 0.003	< 0.003	< 0.003	0.049	< 0.003	< 0.003	< 0.003	0.013	0.013
**I4/0-5**	0.015	0.169	< 0.003	0.043	< 0.003	0.099	0.038	0.057	0.042	0.015	< 0.003
**I5/0-5**	0.030	0.863	0.020	0.055	< 0.003	0.025	0.031	0.076	< 0.003	0.101	< 0.003

### Plants

**Fig 3:**
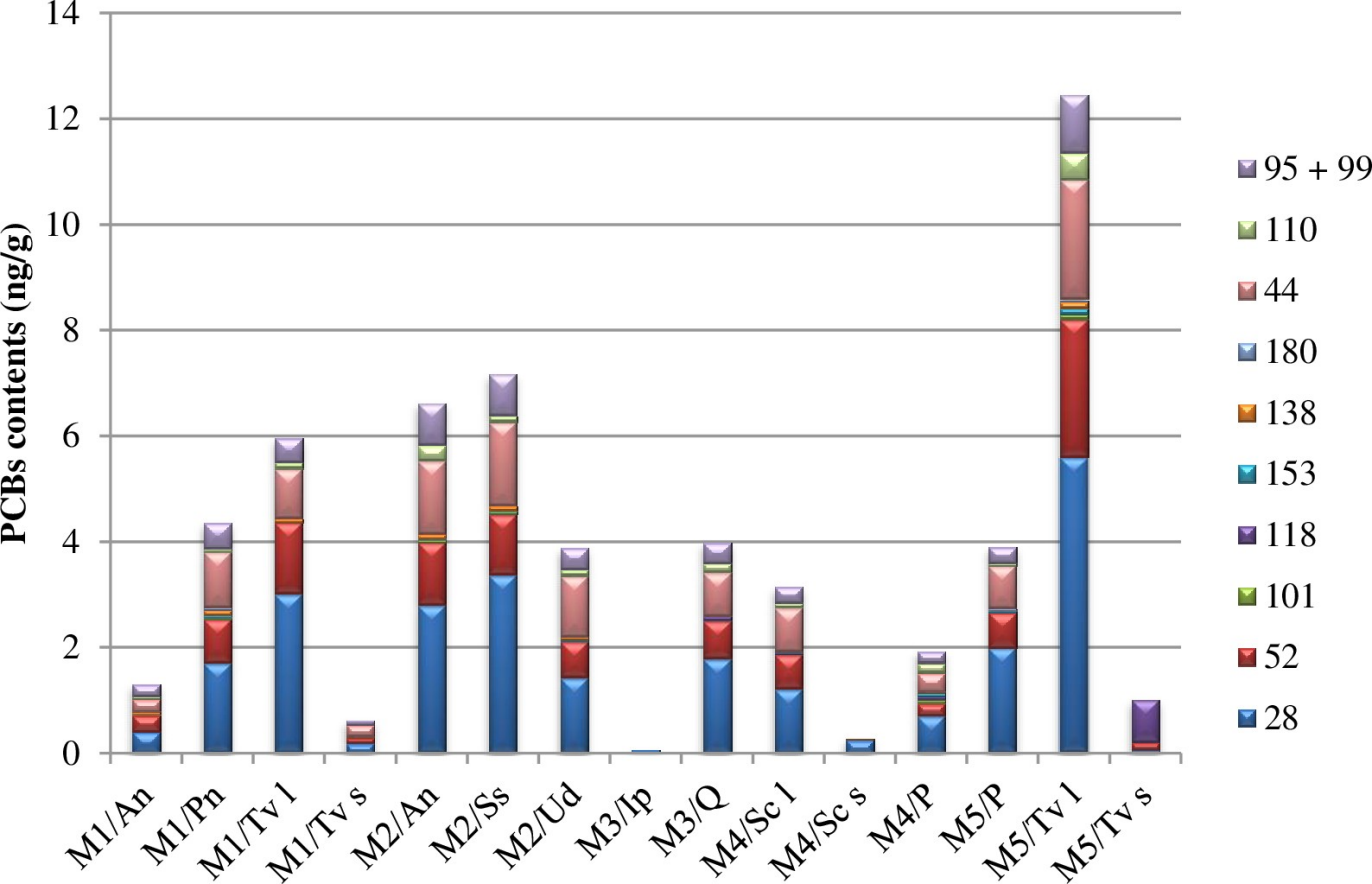
Content of PCBs congeners in plants collected around municipal waste incineration plants; Tv l—*Tanacetum vulgare* leaves, Tv s—*Tanacetum vulgare* stalk, Sc l—*Solidago canadensis* leaves, Sc s—*Solidago canadensis* stalk.

**Fig 4:**
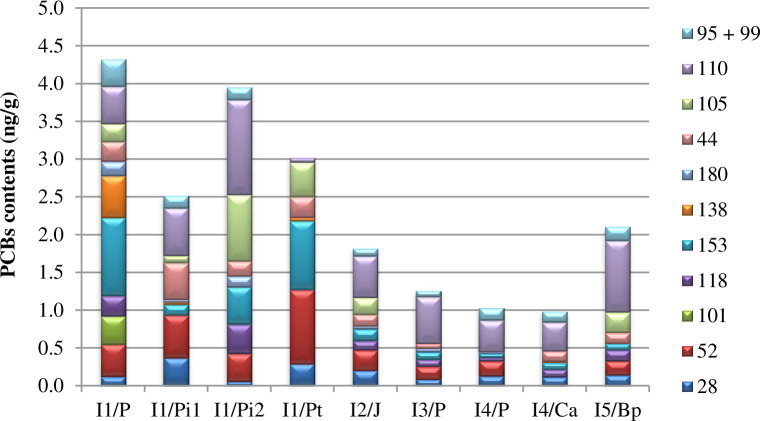
Contents of PCBs congeners in plants collected around industrial waste incineration plants, Pi1—*Pinus L*. one-year-old needles Pi2—*Pinus L*. two-year-old needles.

Plants from test areas around MWI showed significant accumulation of congeners: 28, 52, and 44 (Fig 2). The highest PCBs contents were determined in the leaves of *Tanacetum vulgare* (study area M5). In Figs 3 and 4, on the x-axis, the test plot number is indicated before the slash, and the symbol of the tested plant is provided after the slash. Total PCBs content in leaves of *Tanacetum vulgare* was over 12-fold higher than that in stems of *Tanacetum vulgare* in (M5). On the other hand, in plants from test area M1, these differences were almost six-fold lower. Similarly, in the case of *Solidago canadensis*, the leaves contained much more PCBs than stems. The highest total values of tested PCBs content (Fig 3) after *Tanacetum vulgare* were recorded in leaves of *Acer negundo* and *Salix sepulcralis* (M2). The highest accumulation of PCBs occurred in tall plants and plants with a large leaf area, suggesting that the main source of PCBs in the plants was aerial PCBs deposition. No effect of wind direction or tested distances from the incinerator on PCBs content in plants was observed. The lack of such dependence results from the fact that most of the analysed samples were annual plants, and in the case of trees only leaves, which are also annual, were analysed. During such a short period of exposure, only a small part of contamination will accumulate, while the soil is exposed to contamination over many years leading to the accumulation of more PCBs.

Fig 4 shows the relationship between the content of PCBs congeners in plants harvested from around IWI, depending on the place of collection. In the case of *Pinus L*., one-year-old needles (symbol Pi1) and two-year-old needles (Pi2) were analysed.

The highest totals of PCBs content in plants were found at site I1, corresponding to the highest accumulation of these compounds in soils. Like in the case of soils, total values of tested PCBs content in plants are arranged in the following series: I1 > I5 > I2 > I3 > I4. PCBs content in two-year-old *Pinus L*. needles was higher than that in annual needles, indicating bioaccumulation of PCBs in plants. The total content of tested PCBs in two-year-old pine needles collected at the industrial waste incineration plant was approximately 4 ng/g. In China, in the Dalian urban area, the total content of 209 PCBs congeners in pine needles was 4.4 ng/g on average [28]. In Finland, where pine needles were sampled around a paper industry waste incineration plant, total PCBs congeners 105, 138, 153 were determined to range between 0.6 and 3.0 ng/g [29]. Like in the case of plants from around the municipal waste incinerator, no evident impact of wind direction and distance from the incinerator on PCBs accumulation in the plants was recorded.

### Biological accumulation coefficient

Biological indices can be helpful in the assessment of pollution with PCBs, as well as the accumulation and interaction of PCBs in the environment. The Biological Accumulation Coefficient (BAC) expresses the ratio of PCBs concentration in plants to its weighted average concentration in the soil (0-3 cm). The indicator was calculated for plant stems and for small plants shielded by trees in order to minimize the impact of PCBs deposition from the air. The index was calculated according to the following formula [30]:

BAC=PCBplant/PCBsoil(0−30cm)

PCBs contents in plants and soil below the detection limit were not taken into account for the BAC calculation. It is assumed that compounds for which the bioaccumulation factor is greater than 1 are accumulated [31]. Values of BAC coefficients (Table 6) for MWI showed limited possibilities of intake of compounds belonging to hexa-CB and hepta-CB homologues (PCB 138, PCB 153, PCB 180). In the case of tri-CB and tetra-CB homologues, the values of BAC coefficients significantly exceeded the value of 1.

**Table 6:** Values of BAC for plants from the municipal waste incinerator.

Plot No./plant	PCBs congeners									
	28	52	101	118	153	138	180	44	110	95 + 99
**M1/Pn**	41.92	20.60	0.83	-	0.98	2.37	1.24	26.10	1.51	12.06
**M1/Tv s**	4.63	2.38	-	-	-	0.57	-	5.66	-	2.02
**M2/Ud**	68.98	33.18	-	-	1.52	2.64	-	55.70	5.89	20.04
**M3/Ip**	5.16	-	-	-	-	-	-	-	-	-
**M4/Sc s**	16.17	-	-	-	-	1.39	-	-	-	-
**M4/P**	45.62	14.90	4.35	4.42	3.84	1.78	-	22.81	11.90	14.76
**M5/P**	63.65	21.25	-	-	1.01	-	1.28	25.93	1.56	9.96
**M5/Tv s**	1.74	4.70	-	25.98	-	-	-	-	-	-

Where [–] means not calculated due to PCBs content below the detection limit.

Values of BAC coefficients (Table 7) for IWI showed high intake of PCB 52 (tetra-CB) and PCB 110 (penta-CB). The plants were not washed before PCBs determination. Therefore, high BAC values for PCB 110 are due to the significant content of PCBs in plants, which in turn may be the result of PCBs deposition on plants.

**Table 7:** Values of BAC for plants from the industrial waste incinerator.

Plot No./plant	PCBs congener										
	28	52	101	118	153	138	180	44	105	110	95 + 99
**I1/P**	8.49	30.48	26.67	19.07	74.29	39.43	13.34	19.01	16.84	35.17	25.89
**I3/P**	11.86	24.85	-	14.03	14.64	-	6.21	9.66	-	90.75	12.06
**I4/P**	8.41	13.22	-	2.99	3.90	-	1.10	-	-	28.17	10.69

Where [–] means not calculated due to PCBs content below the detection limit.

### Statistical analysis

The relationships between the content of PCBs congeners in soil and soil pH were calculated. The results showed no statistically significant relationships.

## Conclusions

Among the analysed PCBs congeners, congeners 52, 44, and 110 were accumulated in the topsoil layers from the areas around MWI to the highest degree. In IWI soils, these were congeners 28, 52, and 110. In the case of plants collected around MWI, congeners 28, 52, and 44 were dominant, and in plants from around IWI–congeners 110 and 153. The differences in the dominating congeners most likely result from the combustion of different materials in both incinerators. In the case of MWI, PCBs were observed to move to deeper soil layers, with the highest accumulation of PCBs determined in the layer 20-30 cm due to the loose structure of the analysed soils. In soils around IWI, PCBs did not move to deeper layers, and the largest accumulation of PCBs occurred in the 0-5 cm layer. The influence of wind direction on the accumulation of PCBs in the soil from MWI was not visible. In soils collected on the leeward side of IWI, the accumulation of PCBs was higher than that in soils on the windward side. The dependence of the accumulation of PCBs in the soil on the distance from the emission source, namely the waste incinerator, was observed for samples from the area around MWI and IWI. For plants from around both waste incinerators plants, the effect of wind direction and distance from the incinerator on PCBs accumulation was not evident. The highest accumulation of PCBs occurred in plants with a large leaf area, most likely as a result of aerial deposition. The highest accumulation of PCBs near MWI was recorded in *Tanacetum vulgare* leaves (12.45 ng/g), and near IWI in the grass (4.3 ng/g). In the case of soils, the accumulation of PCBs in the top layers near both incinerators was similar, reaching approximately 3 ng/g. The accumulation of PCBs in plants from MWI was 2-17 times higher than their accumulation in soils. Nonetheless, the accumulation of these compounds in plants around IWI was approximately 2 times higher than in soils.

## Supporting information

S1 Table (DOCX)

S2 Table (DOCX)

S3 Table (DOCX)

S4 Table (DOCX)

## References

[1] Dobson S, Bilthoven GJ van E. Environmental Health Criteria 140 Polichlorinated Biphenyls and Terphenyls. Second Geneva: World Health Organization; 1993. 100–108 p.

[2] WHO. IPCS INTERNATIONAL PROGRAMME ON CHEMICAL SAFETY Health and Safety Guide No. 68 POLYCHLORINATED BIPHENYLS (PCBs) AND POLYCHLORINATED TERPHENYLS (PCTs) HEALTH AND SAFETY GUIDE. WHO 1992 Available from: http://www.inchem.org/documents/hsg/hsg/hsg68.htm#SubSectionNumber:2.5.2.

[3] Larry WR, Larry GH. PCBs: Recent Advances in Environmental Toxicology and Health Effects. Lexington, KY: The University Press of Kentucky; 2001. 185–192 p.

[4] Erickson MD. Introduction: PCB Properties, Uses, Occurrence, and Regulatory History. 2001. Available from: https://mitchelldericksonassociates.files.wordpress.com/2016/09/erickson-pcb-propertiesuse-robertson-book-2000.pdf.

[5] Liu X, Fiedler H, Gong W, Wang B, Yu G. Potential sources of unintentionally produced PCB, HCB, and PeCBz in China: A preliminary overview. Front. Environ. Sci. Eng. 2018; 12 (6): 1 10.1007/s11783-018-1036-9

[6] Bartlett PW, Isaksson E, Hermanson MH. ‘New’ unintentionally produced PCBs in the Arctic. Emerg Contam. 2019; 5: 9–14.

[7] Sakai S, Hiraoka M, Takeda N, Shioxaki K. Behavior of Coplanar PCBs and PCNs in Oxidative Conditions of Municipal Waste Incineration. Chemosphere 1996; 32 (1): 79–88.

[8] Jansson S, Lundin L, Grabic R. Characterisation and fingerprinting of PCBs in flue gas and ash from waste incineration and in technical mixtures. Chemosphere. 2011; 85(3): 509–15. 10.1016/j.chemosphere.2011.08.012 21885088

[9] Shi D, Ma J, Wang H, Wang P, Hu C, Zhang J, et al Detoxification of PCBs in fly ash from MSW incineration by hydrothermal treatment with composite silicon‑aluminum additives and seed induction. Fuel Process Technol. 2019; 195, 106157 10.1016/j.fuproc.2019.106157

[10] Xu P, Wu L, Chen Y, Xu D, Wang X, Shen H, et al High intake of persistent organic pollutants generated by a municipal waste incinerator by breastfed infants. Environ Pollut. 2019; 250: 662–8. 10.1016/j.envpol.2019.04.069 31035148

[11] Wang MS, Chen SJ, Huang KL, Lai YC, Chang-Chien GP, Tsai JH, et al Determination of levels of persistent organic pollutants (PCDD/Fs, PBDD/Fs, PBDEs, PCBs, and PBBs) in atmosphere near a municipal solid waste incinerator. Chemosphere. 2010; 80(10):1220–6. 10.1016/j.chemosphere.2010.06.007 20598339

[12] Güzel B, Canli O, Dede Ş. et al Assessment of PCDD/F and dioxin-like PCB levels in environmental and food samples in the vicinity of IZAYDAS waste incinerator plant (WIP): from past to present. Environ Sci Pollut Res 2020; 27: 13902–13914. 10.1007/s11356-020-07995-y 32036522

[13] Ikonomou MG, Sather P, Oh JE, Choi WY, Chang YS. PCB levels and congener patterns from Korean municipal waste incinerator stack emissions. Chemosphere. 2002; 49(2): 205–216 10.1016/s0045-6535(02)00102-9 12375867

[14] Ling YC, Hou PC c. A Taiwanese study of 2,3,7,8-substituted PCDD/DFs and coplanar PCBs in fly ashes from incinerators. J Hazard Mater. 1998; 58(1–3): 83–91.

[15] Gedik K, Imamoglu I. A preliminary investigation of the environmental impact of a thermal power plant in relation to PCB contamination. Environ Sci Pollut Res. 2011;18(6): 968–77. 10.1007/s11356-010-0430-z 21287286

[16] Lee RGM, Coleman P, Jones JL, Jones KC, Lohmann R. Emission factors and importance of PCDD/Fs, PCBs, PCNs, PAHs and PM 10 from the domestic burning of coal and wood in the U.K. Environ Sci Technol. 2005; 39(6): 1436–47. 10.1021/es048745i 15819195

[17] Ishikawa Y, Noma Y, Yamamoto T, Mori Y, Sakai S ichi. PCB decomposition and formation in thermal treatment plant equipment. Chemosphere. 2007;67(7): 1383–93. 10.1016/j.chemosphere.2006.10.022 17134732

[18] Dyke PH, Foan C, Fiedler H. PCB and PAH releases from power stations and waste incineration processes in the UK. Chemosphere. 2003; 50(4): 469–480. 10.1016/s0045-6535(02)00627-6 12685746

[19] Kim KS, Hong KH, Ko YH, Kim MG. Emission characteristics of pcdd/fs, pcbs, chlorobenzenes, chlorophenols, and pahs from polyvinylchloride combustion at various temperatures. J Air Waste Manag Assoc. 2004; 54(5): 555–62. 10.1080/10473289.2004.10470925 15149043

[20] Moltó J, Font R, Gálvez A, Muñoz M, Pequenín A. Emissions of polychlorodibenzodioxin/furans (PCDD/Fs), dioxin-like polychlorinated biphenyls (PCBs), polycyclic aromatic hydrocarbons (PAHs), and volatile compounds produced in the combustion of pine needles and cones. Energy and Fuels. 2010; 24(2): 1030–6.

[21] Pandelova M, Lenoir D, Schramm KW. Correlation between PCDD/F, PCB and PCBz in coal/waste combustion. Influence of various inhibitors. Chemosphere. 2006; 62(7): 1196–205. 10.1016/j.chemosphere.2005.07.068 16194559

[22] Degrendele C, Fiedler H, Kočan A, Kukučka P, Přribylová P, Prokeš R, et al Multiyear levels of PCDD/Fs, dl-PCBs and PAHs in background air in central Europe and implications for deposition. Chemosphere.2020 240: 124852 10.1016/j.chemosphere.2019.124852 31542585

[23] Dias-Ferreira C, Pato RL, Varejão JB, Tavares AO, Ferreira AJD. Heavy metal and PCB spatial distribution pattern in sediments within an urban catchment—contribution of historical pollution sources. J Soils Sediments.2016 16: 2594–2605. 10.1007/s11368-016-1542-y

[24] Adeyinka GC, Moodley B. Effect of aqueous concentration of humic acid on the sorption of polychlorinated biphenyls onto soil particle grain sizes. Journal of Soils and Sediments. 2019; 19:1543–1553. 10.1007/s11368-018-2147-4

[25] Gabryszewska M, Gworek B, Garlej B. PCB content in soil and plants along routes with high traffic intensity. Desalin WATER Treat. 2018; 117: 211–220. 10.5004/dwt.2018.22398

[26] Bartlett PW, Isaksson E, Hermanson MH. ‘New’ unintentionally produced PCBs in the Arctic. Emerg Contam. 2019; 5: 9–14. 10.1016/j.emcon.2018.12.004

[27] Baran A, Mierzwa-hersztek M, Urbaniak M, Gondek K, Tarnawski M. An assessment of the concentrations of PCDDs/Fs in contaminated bottom sediments and their sources and ecological risk. J Soils Sediments. 2019; 20: 2588–2597. 10.1007/s11368-019-02492-3

[28] Chen J, Zhao H, Gao L, Henkelmann B, Schramm KW. Atmospheric PCDD/F and PCB levels implicated by pine (Cedrus deodara) needles at Dalian, China. Environ Pollut. 2006; 144(2): 510–5. 10.1016/j.envpol.2006.01.039 16545895

[29] Sinkkonen S, Kämäräinen N, Paasivirta J, Lammi R. PCDDs, PCDFs, PCDTs, PCBs and some other organochlorine compounds in pine needles exposed to pulp and paper mill emissions and effects of waste combustion on the concentrations. Chemosphere. 1997; 35(10): 2193–202.

[30] Gworek B, Dmuchowski W, Koda E, Marecka M, Baczewska AH, Bragoszewska P, et al Impact of the municipal solid waste lubna landfill on environmental pollution by heavy metals. Water. 2016; 8, 470 10.3390/w8100470

[31] Whitfield Åslund ML, Rutter A, Reimer KJ, Zeeb BA. The effects of repeated planting, planting density, and specific transfer pathways on PCB uptake by Cucurbita pepo grown in field conditions. Sci Total Environ. 2008; 405(1–3): 14–25. 10.1016/j.scitotenv.2008.07.066 18786697

