# Supplementary material for: Impact of municipal and industrial waste incinerators on PCBs content in the environment
Source: PLoS One. 2020 Nov 19;15(11):e0242698. doi: 10.1371/journal.pone.0242698 (PMC7676720; doi:10.1371/journal.pone.0242698)
Supplement: S1 Table — (DOCX) [file pone.0242698.s001.docx]

| **Plot No./**  **depth (cm)** | **PCBs congeners content (ng/g)** | | | | | | | | | | | **pH in H_2_O** | **pH in KCl** | **TOC (%)** |
| --- | --- | --- | --- | --- | --- | --- | --- | --- | --- | --- | --- | --- | --- | --- |
|  | **28** | **52** | **101** | **118** | **153** | **138** | **180** | **44** | **105** | **110** | **95 + 99** |  |  |  |
| **M1/0-5** | < 0.003 | 0.167 | 0.012 | 0.043 | < 0.003 | < 0.003 | 0.061 | 0.232 | 0.200 | 1.982 | < 0.003 | 7.75 | 7.37 | 2.18 |
| **M1/5-10** | 0.057 | 0.203 | < 0.003 | 0.019 | < 0.003 | 0.005 | 0.058 | 0.137 | 0.090 | 0.756 | < 0.003 | 7.87 | 7.48 | 2.37 |
| **M1/10-20** | 0.087 | 0.311 | < 0.003 | 0.031 | < 0.003 | < 0.003 | 0.089 | 0.206 | 0.086 | 0.727 | 0.034 | 7.85 | 7.5 | 3.47 |
| **M1/20-30** | 0.006 | 0.381 | 0.021 | 0.065 | < 0.003 | 0.021 | 0.336 | 0.265 | 0.352 | 1.489 | 0.080 | 8.2 | 7.83 | 2.08 |
| **M2/0-5** | 0.028 | 0.182 | 0.080 | 0.168 | 0.179 | < 0.003 | < 0.003 | 0.281 | 0.086 | 0.193 | 0.146 | 6.79 | 6.14 | 9.19 |
| **M2/5-10** | 0.018 | 0.184 | 0.087 | 0.101 | 0.163 | < 0.003 | < 0.003 | 0.473 | 0.091 | 0.300 | 0.112 | 6.74 | 5.97 | 12 |
| **M2/10-20** | 0.029 | 0.222 | 0.144 | 0.107 | 0.164 | < 0.003 | < 0.003 | 0.299 | 0.131 | 0.328 | 0.189 | 6.86 | 5.94 | 13 |
| **M2/20-30** | 0.010 | 0.405 | 0.135 | 0.065 | 0.078 | < 0.003 | < 0.003 | 0.644 | 0.053 | 0.266 | 0.071 | 6.8 | 6.01 | 10 |
| **M3/0-5** | 0.010 | 0.037 | 0.054 | 0.056 | 0.060 | < 0.003 | 0.041 | < 0.003 | 0.037 | 0.186 | 0.042 | 5.05 | 3.98 | 0.79 |
| **M3/5-10** | 0.012 | 0.011 | < 0.003 | 0.020 | < 0.003 | < 0.003 | 0.054 | < 0.003 | 0.022 | 0.018 | < 0.003 | 5.18 | 4.05 | 0.24 |
| **M3/10-20** | 0.012 | 0.040 | < 0.003 | < 0.003 | 0.006 | < 0.003 | < 0.003 | < 0.003 | 0.022 | < 0.003 | 0.026 | 5.44 | 4.24 | 0.26 |
| **M3/20-30** | 0.013 | < 0.003 | < 0.003 | < 0.003 | < 0.003 | < 0.003 | 0.021 | < 0.003 | < 0.003 | < 0.003 | 0.047 | 5.73 | 4.39 | 0.1 |
| **M4/0-5** | 0.007 | 0.044 | < 0.003 | < 0.003 | 0.041 | < 0.003 | 0.039 | < 0.003 | < 0.003 | 0.040 | 0.014 | 5.7 | 4.3 | 1.18 |
| **M4/5-10** | 0.013 | 0.031 | < 0.003 | 0.015 | 0.101 | < 0.003 | 0.033 | < 0.003 | < 0.003 | 0.053 | < 0.003 | 5.29 | 3.97 | 0.96 |
| **M4/10-20** | 0.019 | 0.042 | < 0.003 | 0.072 | 0.078 | < 0.003 | 0.021 | < 0.003 | < 0.003 | 0.050 | < 0.003 | 5.25 | 4.01 | 0.88 |
| **M4/20-30** | 0.012 | 0.090 | < 0.003 | 0.048 | 0.067 | < 0.003 | 0.015 | 0.015 | 0.017 | 0.042 | < 0.003 | 5.34 | 4.09 | 0.81 |
| **M1/0-5** | 0.036 | 0.079 | 0.052 | 0.041 | 0.099 | < 0.003 | < 0.003 | 0.306 | 0.022 | 0.144 | 0.025 | 5.76 | 4.97 | 14.2 |
| **M1/5-10** | 0.024 | 0.126 | 0.040 | 0.055 | 0.050 | < 0.003 | < 0.003 | 0.508 | < 0.003 | 0.072 | 0.038 | 5.61 | 4.78 | 12.2 |
| **M1/10-20** | 0.018 | 0.242 | 0.039 | 0.061 | < 0.003 | < 0.003 | < 0.003 | 0.868 | 0.016 | < 0.003 | 0.056 | 5.66 | 4.86 | 11.3 |
| **M1/20-30** | 0.046 | 0.469 | 0.164 | 0.026 | < 0.003 | < 0.003 | < 0.003 | < 0.003 | < 0.003 | < 0.003 | 0.120 | 5.79 | 5 | 8.03 |
