# Supplementary material for: Impact of municipal and industrial waste incinerators on PCBs content in the environment
Source: PLoS One. 2020 Nov 19;15(11):e0242698. doi: 10.1371/journal.pone.0242698 (PMC7676720; doi:10.1371/journal.pone.0242698)
Supplement: S2 Table — (DOCX) [file pone.0242698.s002.docx]

| **Plot no./plant** | **PCBs congeners content (ng/g)** | | | | | | | | | |
| --- | --- | --- | --- | --- | --- | --- | --- | --- | --- | --- |
|  | **28** | **52** | **101** | **118** | **153** | **138** | **180** | **44** | **110** | **95 + 99** |
| **M1/An** | 0.407 | 0.313 | < 0.003 | < 0.003 | < 0.003 | 0.058 | < 0.003 | 0.237 | 0.056 | 0.236 |
| **M1/Pn** | 1.698 | 0.834 | 0.034 | < 0.003 | 0.040 | 0.096 | 0.050 | 1.057 | 0.061 | 0.489 |
| **M1/Tv l** | 3.007 | 1.350 | < 0.003 | < 0.003 | < 0.003 | 0.073 | < 0.003 | 0.949 | 0.116 | 0.469 |
| **M1/Tv s** | 0.188 | 0.096 | < 0.003 | < 0.003 | < 0.003 | 0.023 | < 0.003 | 0.229 | < 0.003 | 0.082 |
| **M2/An** | 2.793 | 1.191 | 0.049 | < 0.003 | 0.014 | 0.090 | < 0.003 | 1.394 | 0.289 | 0.805 |
| **M2/Ss** | 3.373 | 1.142 | 0.054 | < 0.003 | 0.016 | 0.089 | < 0.003 | 1.586 | 0.124 | 0.791 |
| **M2/Ud** | 1.426 | 0.686 | < 0.003 | < 0.003 | 0.031 | 0.055 | < 0.003 | 1.151 | 0.122 | 0.414 |
| **M3/Ip** | 0.062 | < 0.003 | < 0.003 | < 0.003 | < 0.003 | < 0.003 | < 0.003 | < 0.003 | < 0.003 | < 0.003 |
| **M3/Q** | 1.785 | 0.720 | < 0.003 | 0.092 | < 0.003 | < 0.003 | < 0.003 | 0.822 | 0.173 | 0.395 |
| **M4/Sc l** | 1.212 | 0.653 | < 0.003 | 0.031 | < 0.003 | < 0.003 | 0.023 | 0.835 | 0.084 | 0.314 |
| **M4/Sc s** | 0.251 | < 0.003 | < 0.003 | < 0.003 | < 0.003 | 0.021 | < 0.003 | < 0.003 | < 0.003 | < 0.003 |
| **M4/P** | 0.707 | 0.231 | 0.067 | 0.068 | 0.060 | 0.028 | < 0.003 | 0.354 | 0.184 | 0.229 |
| **M5/P** | 1.994 | 0.666 | < 0.003 | < 0.003 | 0.032 | < 0.003 | 0.040 | 0.813 | 0.049 | 0.312 |
| **M5/Tv l** | 5.585 | 2.619 | 0.095 | < 0.003 | 0.112 | 0.136 | 0.036 | 2.266 | 0.497 | 1.104 |
| **M5/Tv s** | 0.055 | 0.147 | < 0.003 | 0.814 | < 0.003 | < 0.003 | < 0.003 | < 0.003 | < 0.003 | < 0.003 |
