# Supplementary material for: Impact of municipal and industrial waste incinerators on PCBs content in the environment
Source: PLoS One. 2020 Nov 19;15(11):e0242698. doi: 10.1371/journal.pone.0242698 (PMC7676720; doi:10.1371/journal.pone.0242698)
Supplement: S3 Table — (DOCX) [file pone.0242698.s003.docx]

| **Plot No./**  **depth (cm)** | **PCBs congeners content (ng/g)** | | | | | | | | | | | **pH in H_2_O** | **pH in KCl** | **TOC (%)** |
| --- | --- | --- | --- | --- | --- | --- | --- | --- | --- | --- | --- | --- | --- | --- |
|  | **28** | **52** | **101** | **118** | **153** | **138** | **180** | **44** | **105** | **110** | **95 + 99** |  |  |  |
| **I1/0-5** | 0.178 | 0.439 | 0.210 | 0.047 | 2.333 | < 0.003 | 0.384 | 0.163 | 0.026 | 0.404 | 0.158 | 3.6 | 3.00 | 35.3 |
| **I1/5-10** | 0.053 | 0.199 | 0.046 | 0.000 | < 0.003 | 0.044 | 0.325 | < 0.003 | < 0.003 | 0.189 | 0.168 | 3.93 | 3.33 | 8.07 |
| **I1/10-20** | 0.310 | 0.511 | 0.325 | 0.147 | 1.584 | 0.176 | 0.511 | 0.253 | 0.050 | 0.420 | 0.210 | 3.65 | 3.13 | 35.6 |
| **I1/20-30** | 0.125 | 0.388 | 0.102 | 0.059 | < 0.003 | 0.073 | 0.310 | 0.170 | 0.054 | 0.240 | 0.113 | 3.83 | 3.3 | 21.4 |
| **I2/0-5** | 0.018 | 0.051 | 0.020 | < 0.003 | < 0.003 | < 0.003 | 0.028 | < 0.003 | 0.059 | 0.027 | 0.010 | 4.3 | 3.53 | 3.25 |
| **I2/5-10** | 0.013 | 0.030 | < 0.003 | < 0.003 | < 0.003 | < 0.003 | < 0.003 | < 0.003 | < 0.003 | 0.006 | 0.014 | 5.17 | 4.02 | 0.34 |
| **I2/10-20** | 0.026 | 0.040 | < 0.003 | < 0.003 | < 0.003 | 0.036 | < 0.003 | < 0.003 | 0.024 | < 0.003 | < 0.003 | 4.7 | 3.85 | 0.58 |
| **I2/20-30** | 0.007 | < 0.003 | 0.015 | < 0.003 | < 0.003 | 0.006 | < 0.003 | < 0.003 | 0.013 | 0.035 | 0.015 | 4.73 | 3.94 | 0.31 |
| **I3/0-5** | 0.016 | < 0.003 | < 0.003 | < 0.003 | < 0.003 | < 0.003 | < 0.003 | 0.020 | < 0.003 | < 0.003 | < 0.003 | 4.95 | 3.8 | 0.1 |
| **I3/5-10** | 0.037 | 0.139 | 0.034 | < 0.003 | 1.755 | < 0.003 | 0.065 | 0.114 | 0.059 | 0.089 | 0.061 | 7.44 | 7.52 | 2.48 |
| **I3/10-20** | 0.005 | 0.033 | 0.027 | 0.040 | < 0.003 | < 0.003 | 0.010 | 0.019 | < 0.003 | 0.015 | < 0.003 | 7.57 | 7.6 | 2.05 |
| **I3/20-30** | 0.010 | 0.068 | < 0.003 | 0.029 | < 0.003 | < 0.003 | < 0.003 | < 0.003 | < 0.003 | 0.015 | 0.016 | 7.74 | 7.73 | 0.35 |
| **I4/0-5** | 0.011 | 0.049 | < 0.003 | < 0.003 | < 0.003 | 0.061 | 0.021 | < 0.003 | < 0.003 | 0.016 | 0.006 | 7.86 | 7.83 | 0.27 |
| **I5/0-5** | 0.013 | 0.236 | 0.035 | 0.081 | 1.823 | 0.048 | 0.086 | 0.065 | < 0.003 | 0.160 | 0.041 | 7.48 | 7.1 | 5.43 |
| **I1/0-5** | 0.048 | 0.117 | 0.032 | 0.014 | < 0.003 | 0.044 | 0.061 | 0.015 | < 0.003 | 0.171 | 0.019 | 7.68 | 7.26 | 1.73 |
| **I1/5-10** | < 0.003 | < 0.003 | < 0.003 | < 0.003 | < 0.003 | 0.040 | < 0.003 | 0.039 | < 0.003 | 0.194 | 0.006 | 7.8 | 7.41 | 1.29 |
| **I1/10-20** | 0.013 | 0.084 | 0.014 | 0.029 | < 0.003 | 0.074 | 0.045 | 0.032 | 0.029 | 0.113 | 0.007 | 7.77 | 7.38 | 2.63 |
| **I1/20-30** | 0.014 | < 0.003 | 0.022 | < 0.003 | < 0.003 | 0.017 | < 0.003 | 0.017 | < 0.003 | 0.067 | 0.014 | 7.96 | 7.7 | 2.67 |
| **I2/0-5** | 0.007 | 0.092 | < 0.003 | 0.016 | 0.158 | < 0.003 | < 0.003 | < 0.003 | 0.026 | 0.048 | < 0.003 | 7.88 | 7.72 | 5.05 |
| **I2/5-10** | 0.011 | 0.070 | 0.029 | 0.054 | < 0.003 | 0.040 | 0.174 | 0.027 | 0.079 | 0.185 | 0.034 | 7.57 | 6.93 | 2.06 |
| **I2/10-20** | 0.012 | 0.078 | 0.019 | < 0.003 | < 0.003 | 0.079 | 0.073 | 0.015 | 0.072 | 0.342 | 0.031 | 7.46 | 7.17 | 1.1 |
| **I2/20-30** | 0.005 | 0.058 | < 0.003 | 0.011 | < 0.003 | 0.055 | 0.039 | 0.006 | 0.071 | 0.132 | 0.035 | 7.5 | 7.11 | 1.12 |
| **I3/0-5** | 0.015 | 0.081 | < 0.003 | < 0.003 | < 0.003 | 0.123 | 0.044 | 0.012 | 0.049 | 0.411 | < 0.003 | 7.58 | 7.14 | 0.9 |
| **I3/5-10** | 0.004 | 0.016 | < 0.003 | < 0.003 | < 0.003 | 0.049 | < 0.003 | < 0.003 | < 0.003 | 0.013 | 0.013 | 7.56 | 7.17 | 0.19 |
| **I3/10-20** | 0.014 | 0.051 | 0.015 | < 0.003 | < 0.003 | 0.039 | < 0.003 | 0.009 | < 0.003 | < 0.003 | 0.022 | 7.81 | 7.45 | 1.35 |
| **I3/20-30** | 0.011 | 0.025 | < 0.003 | 0.024 | 0.105 | 0.035 | < 0.003 | 0.007 | 0.010 | 0.021 | 0.011 | 8 | 7.56 | 1.56 |
| **I4/0-5** | 0.015 | 0.169 | < 0.003 | 0.043 | < 0.003 | 0.099 | 0.038 | 0.057 | 0.042 | 0.015 | < 0.003 | 7.28 | 7.38 | 3.69 |
| **I5/0-5** | 0.030 | 0.863 | 0.020 | 0.055 | < 0.003 | 0.025 | 0.031 | 0.076 | < 0.003 | 0.101 | < 0.003 | 8.17 | 7.99 | 3.97 |
