# Supplementary material for: Impact of municipal and industrial waste incinerators on PCBs content in the environment
Source: PLoS One. 2020 Nov 19;15(11):e0242698. doi: 10.1371/journal.pone.0242698 (PMC7676720; doi:10.1371/journal.pone.0242698)
Supplement: S4 Table — (DOCX) [file pone.0242698.s004.docx]

| **Plot no./plant** | **PCBs congeners content (ng/g)** | | | | | | | | | | |
| --- | --- | --- | --- | --- | --- | --- | --- | --- | --- | --- | --- |
|  | **28** | **52** | **101** | **118** | **153** | **138** | **180** | **44** | **105** | **110** | **95 + 99** |
| **I1/P** | 0.119 | 0.427 | 0.373 | 0.267 | 1.040 | 0.552 | 0.187 | 0.266 | 0.236 | 0.492 | 0.362 |
| **I1/Pt** | 0.365 | 0.567 | < 0.003 | < 0.003 | 0.144 | 0.028 | 0.035 | 0.487 | 0.089 | 0.636 | 0.169 |
| **I1/Pi1** | 0.055 | 0.370 | < 0.003 | 0.389 | 0.491 | < 0.003 | 0.144 | 0.201 | 0.875 | 1.260 | 0.167 |
| **I1/Pi2** | 0.289 | 0.979 | < 0.003 | < 0.003 | 0.911 | 0.041 | < 0.003 | 0.274 | 0.456 | 0.061 | < 0.003 |
| **I2/J** | 0.200 | 0.270 | < 0.003 | 0.124 | 0.155 | < 0.003 | 0.039 | 0.153 | 0.221 | 0.551 | 0.102 |
| **I3/P** | 0.081 | 0.170 | < 0.003 | 0.096 | 0.100 | < 0.003 | 0.042 | 0.066 | < 0.003 | 0.620 | 0.082 |
| **I4/P** | 0.126 | 0.198 | < 0.003 | 0.045 | 0.058 | < 0.003 | 0.017 | < 0.003 | < 0.003 | 0.423 | 0.160 |
| **I4/Ca** | 0.115 | < 0.003 | < 0.003 | 0.102 | 0.088 | 0.012 | < 0.003 | 0.140 | < 0.003 | 0.384 | 0.146 |
| **I5/Bp** | 0.139 | 0.187 | < 0.003 | 0.148 | 0.086 | 0.030 | < 0.003 | 0.142 | 0.267 | 0.952 | 0.188 |
